# Integrated microarray-based and single-cell transcriptomic analyses reveal cell adhesion-related fibrotic remodeling signatures in heart failure

**DOI:** 10.3389/fmolb.2026.1929610

**Published:** 2026-09-22

**Authors:** Shun Zhang, Le Li, Zuohua Li, Xiaojun Wang, Juan Zhou, Qing Lyu

**Affiliations:** 1 Department of Cardiology, Taikang Tongji (Wuhan) Hospital, Wuhan, Hubei, China; 2 Department of Orthopedics, Taikang Tongji (Wuhan) Hospital, Wuhan, China; 3 Department of Orthopedics Trauma and Microsurgery, Zhongnan Hospital, Wuhan University, Wuhan, China

**Keywords:** biomarker, cell adhesion, fibroblast remodeling, heart failure, single-cell transcriptomic analysis

## Abstract

**Background:**

Heart failure (HF) is characterized by impaired cardiac pumping function and progressive remodeling. Cell adhesion is essential for tissue integrity and the regulation of inflammation and fibrosis. This study aimed to identify key cell adhesion-related genes (CARGs) and explore their roles in HF.

**Methods:**

HF-related transcriptomic datasets (GSE1621, GSE5500, GSE36074, GSE18224, GSE308859, and GSE116250) were obtained from GEO. Differentially expressed genes were intersected with cell adhesion-related genes from MSigDB to identify CARGs. Functional enrichment and protein-protein interaction analyses were performed. A CARG-based CelladhesionScore was constructed to evaluate its discriminatory value and associated pathways. Key CARGs were identified using CytoHubba. Single-cell transcriptomic analysis was conducted to assess fibroblast states and gene dynamics. GSE36074 and a transverse aortic constriction (TAC) mouse model were used for validation by qPCR, Western blotting, and immunohistochemistry.

**Results:**

Forty-five differentially expressed CARGs were identified and were mainly enriched in cell adhesion, extracellular region, and ECM-receptor interaction pathways. The CelladhesionScore distinguished HF samples from controls, while the high-CelladhesionScore group showed enrichment of fibrosis-related pathways. *Col5a1*, *Col6a1*, and *Fbn1* were identified as key CARGs. Single-cell analysis showed that these genes were predominantly expressed in fibroblasts and associated with fibroblast-state changes and ECM remodeling. Their upregulation was consistently validated in external datasets and the TAC mouse model.

**Conclusion:**

*Col5a1*, *Col6a1*, and *Fbn1* are key CARGs associated with HF and fibroblast-mediated ECM remodeling. These findings provide new insights into cell adhesion-related molecular alterations in HF and may facilitate the identification of candidate biomarkers and therapeutic targets.

## Introduction

Heart failure (HF) is a clinical syndrome caused by structural or functional abnormalities of the heart, leading to impaired filling of blood and an inability to meet the metabolic demands of body tissues at rest or during activity (Bozkurt et al., 2021). Heart failure predominantly affects older adults, and its prevalence increases markedly with advancing age, making the ageing population a major contributor to the growing global burden of heart failure (Khan et al., 2024). In the progression of heart failure, compensatory mechanisms lead to cardiomyocyte hypertrophy, while myocardial injury induces inflammatory responses. Meanwhile, the presence of myocardial stretch and inflammation activates fibroblasts, thereby contributing to the progression of myocardial fibrosis (Frantz et al., 2022). These pathological changes are interconnected and mutually reinforcing, collectively contributing to the progressive deterioration of cardiac structure and function. Despite advances in heart failure management, the effective control of adverse cardiac remodeling remains a critical and unresolved challenge. In the early stages of HF, structural deterioration may be reversible through timely control of underlying conditions such as hypertension and myocardial ischemia. Additionally, pharmacological treatments, including angiotensin-converting enzyme inhibitors (ACEIs) (Pfeffer et al., 1992), angiotensin receptor blockers (ARBs) (Pfeffer et al., 2022), and β-adrenergic blockers (Clemente-Moragon et al., 2020), have demonstrated efficacy in mitigating or even reversing cardiac remodeling. However, in the advanced stages of heart failure, prolonged cardiac overload results in extensive cardiomyocyte death, severe myocardial fibrosis, and significant ventricular dilation (Machackova et al., 2006; Gerdes and Capasso, 1995). At this advanced stage, adverse cardiac remodeling can only be slowed, but not fully reversed. Therefore, identifying novel therapeutic targets and biological markers is of critical importance.

Cell adhesion is a critical biological process that maintains tissue architecture, mediates signal transduction, and facilitates immune responses during organismal development (Hynes, 1999).Under various pathological conditions of the heart, alterations in cell–cell adhesion lead to abnormal cardiomyocyte connectivity and impaired signal transduction (Zhao et al., 2019). These changes promote inflammatory responses, and fibrosis, thereby accelerating the progression of adverse cardiac remodeling. Cell adhesion molecules play a key role in initiating and regulating inflammatory responses by mediating the adhesion and rolling of neutrophils on vascular endothelial cells, ultimately facilitating their migration to sites of inflammation (Albelda et al., 1994). When inflammatory cells migrate to the site of inflammation, they activate fibroblasts and stimulate pro-fibrotic signaling pathways such as TGF-β, thereby exacerbating tissue fibrosis (Koivisto et al., 2018; Hintermann and Christen, 2019). Recent studies have indicated that intercellular adhesion molecule-1 is associated with cardiac function in heart failure and atrial fibrillation (Mathew et al., 2024), and that missense mutations in intercellular adhesion molecule-1 are linked to an increased risk of heart failure (Giro et al., 2023).

This study analyzed heart failure-related transcriptomic datasets to identify differentially expressed cell adhesion-related genes (CARGs) and investigated their associated biological processes through enrichment analysis. A CelladhesionScore model was constructed based on the expression profiles of CARGs to evaluate its potential diagnostic value for HF and to explore the molecular pathways associated with the high- and low-risk CelladhesionScore groups in HF samples. The interactions among differentially expressed CARGs were explored through a protein-protein interaction (PPI) network, and key CARGs were identified using multiple algorithms. Single-cell transcriptomic analysis was further performed to investigate the dynamic changes in fibroblasts during HF progression, as well as the cellular distribution and dynamic expression patterns of key CARGs. In addition, key CARGs were validated using an external heart failure-related dataset. Finally, the expression of key CARGs were further verified by qPCR, Western blot, and immunohistochemistry (IHC) staining in the hearts of mice with the transverse aortic constriction (TAC) model.

## Methods

### Data source

The HF-related datasets GSE1621 (mouse), GSE5500 (mouse), GSE36074 (mouse), GSE18224 (mouse), GSE308859 (mouse), and GSE116250 (human) were obtained from the Gene Expression Omnibus (GEO) database (https://www.ncbi.nlm.nih.gov/). The included mouse datasets met the following characteristics: (1) The experimental groups all underwent transverse aortic constriction (TAC) surgery and exhibited signs of heart failure; (2) the samples were derived from cardiac tissue; (3) complete raw mRNA data were provided. The included human dataset met the following characteristics: (1) samples in the experimental groups were obtained from patients with heart failure caused by ischemic heart disease; (2) the samples were derived from cardiac tissue; (3) complete raw mRNA data were provided. GSE1621 is based on the GPL81 platform ([MG_U74Av2] Affymetrix Murine Genome U74A Version 2 Array). GSE5500 is based on GPL1261 platform ([Mouse430_2] Affymetrix Mouse Genome 430 2.0 Array). GSE36074 is based on the GPL1261 platform ([Mouse430_2] Affymetrix Mouse Genome 430 2.0 Array). GSE18224 is based on the GPL1261 platform ([Mouse430_2] Affymetrix Mouse Genome 430 2.0 Array). GSE1621 contains four control group samples and four experimental group samples, in which the hearts of experimental mice were collected on the 10th day after TAC surgery. GSE5500 contains four control group samples and six experimental group samples, in which the hearts of experimental mice were collected on the seventh day after TAC surgery. GSE36074 contains seven control group samples and seven experimental group samples, in which the hearts of experimental mice were collected at the fourth week after TAC surgery. GSE18224 contains eight control group samples and eight experimental group samples, in which the hearts of experimental mice were collected at the ninth week after TAC surgery. GSE116250 is based on the GPL16791 platform (Illumina HiSeq 2500 (*Homo sapiens*)). GSE116250 contains 14 non-HF samples and 13 HF samples caused by ischemic heart disease. The single-cell transcriptomic dataset GSE308859 is based on the GPL24247 platform (Illumina NovaSeq 6000 (*Mus musculus*)) and included four samples: Sham, TAC 2 weeks, TAC 4 weeks, and TAC 6 weeks. In this study, GSE1621 and GSE5500 were used as the analysis datasets, GSE18224 and GSE116250 were used to validate the CelladhesionScore, GSE36074 was used to validate the expression of the key CARGs, and GSE308859 was used for single-cell transcriptomic analysis.

### Identification of DEGs and differential expression CARGs

Differential expression analysis of HF-related datasets was performed using the “Limma” package in R software to identify the differentially expressed genes (DEGs) between the control and experimental groups. The criteria for identifying DEGs were: P-value <0.05 and Fold Change >1.5. 1507 CARGs were selected from the MSigDB website (https://www.gsea-msigdb.org/gsea/msigdb). Information on the selected gene sets is provided in Supplementary Material S4. The intersection of DEGs and CARGs was taken to obtain differentially expressed CARGs. The expression profiles of differentially expressed CARGs in both datasets are provided in Supplementary Material S5.

### Functional and pathway enrichment analysis

The “clusterProfiler” package in R software was used for Gene Ontology (GO) (Gene Ontology Consortium, 2015) analysis and Kyoto Encyclopedia of Genes and Genomes (KEGG) (Kanehisa and Goto, 2000) enrichment analysis. P < 0.05 was considered statistically significant.

### Construction and validation of the CelladhesionScore

The GSE1621 and GSE5500 datasets were merged as the training set, and batch effects after integration were removed using the R package “sva”. The GSE116250 and GSE18224 datasets were used as validation datasets for evaluating the CelladhesionScore. Differentially expressed CARGs were used as the input gene set to calculate the CelladhesionScore by single-sample gene set enrichment analysis (ssGSEA). ssGSEA was selected because it enables the estimation of gene signature activity at the individual-sample level without requiring predefined phenotype labels or model training. This approach is suitable for microarray-based transcriptomic datasets and allows the quantification of cell adhesion-related transcriptional features in each sample. Based on the median CelladhesionScore, samples in the training set were stratified into high- and low-CelladhesionScore groups. Principal component analysis (PCA) was performed to assess the separation between the high- and low-CelladhesionScore groups. Receiver operating characteristic (ROC) curve analysis was performed to evaluate the discriminatory ability of the CelladhesionScore in distinguishing HF samples from control samples in both the training and validation datasets.

### Analysis of biological functional differences and fibrosis-related pathways among CelladhesionScore subtypes in HF

Gene Set Variation Analysis (GSVA) was performed using the Hallmark gene sets from the MSigDB to assess differentially activated pathways between the high-risk and low-risk groups defined by CelladhesionScore in HF samples from the training set. Gene sets associated with fibrosis were selected from public pathway resources (MSigDB, Reactome, and GO), to generate a custom GMT file, which was then used to assess differentially enriched fibrosis-related pathways between the high-risk and low-risk groups. Spearman correlation analysis was performed to evaluate the correlation between CelladhesionScore and fibrosis-related pathways.

### Protein-protein interaction (PPI) analysis and identification of key CARGs

The PPI analysis of differentially expressed CARGs was conducted using the online STRING database. The visualization of the PPI network was achieved using Cytoscape software. The CytoHubba plugin in Cytoscape software was used to identify key CARGs, with three algorithms (MNC, MCC, and DMNC) applied for screening. The intersecting genes identified by the three algorithms were considered key CARGs.

### Expression patterns of key CARGs in the training and validation datasets

An independent samples t-test was performed to assess the differences in key CARGs between the control and experimental groups in the training datasets GSE1621 and GSE5500, and the results were visualized using box plots. Pearson correlation analysis was conducted in the training datasets GSE1621 and GSE5500 to evaluate the correlations among the key CARGs. The expression trends of key CARGs were validated in the HF-related external dataset GSE36074 using independent sample t-tests. P < 0.05 was considered statistically significant.

### Additional exploratory analyses

To explore potential intervention-related targets associated with the key CARGs, immune infiltration analysis, miRNA prediction, and exploratory molecular docking simulations were performed. These findings require further experimental validation. Detailed methods and results are provided in Supplementary Material S1-S3.

### Single-cell RNA sequencing data processing, cell-type annotation, and cell-cell communication analysis

The single-cell RNA sequencing (scRNA-seq) dataset GSE308859 was processed using the R package “Seurat” (version 4.0). For quality control, cells were retained according to the following criteria: 200–6000 detected genes, 500–30000 UMIs, and a mitochondrial gene percentage below 15%. The quality-controlled single-cell expression matrix was normalized using the LogNormalize method with a scale factor of 10,000. Potential doublets were identified separately for each sample using the R package “scDblFinder” (version 1.26.0), and only cells classified as singlets were retained for downstream analysis. Subsequently, the top 3,000 highly variable genes were identified using the vst method, and data scaling was performed based on these highly variable genes. Subsequently, PCA was performed based on the highly variable genes, and the first 25 principal components were selected for cell-cell nearest-neighbor graph construction, unsupervised clustering, and UMAP-based dimensionality reduction and visualization. The clustering resolution was set to 0.2. Marker genes for each subtype were identified using the “FindAllMarkers” function, and differential expression analysis was performed. The filtering criteria were as follows: |log2FC| > 0.5, and adjusted P value <0.05. Major cell types were annotated based on canonical marker genes.

### Fibroblast subclustering and pseudotime analysis

Fibroblasts were subsetted from the final annotated Seurat object and reclustered at a low resolution to preserve broad biological states suitable for downstream trajectory analysis. Based on representative marker gene expression, fibroblasts were annotated into four putative functional states: Resident Fib (Dcn, Lum, Col14a1, Pdgfra), Transitional Fib (Pi16, Cxcl14, Adamts5, Pcsk6), ECM-remodeling Fib (Comp, Col5a1, Col6a1, Fbn1, Pcolce, Lox), and Activated secretory Fib (Wif1, Prg4, Clu, Dkk3, Ecrg4).

Pseudotime trajectory analysis was performed using Monocle2. The fibroblast Seurat object was converted into a Monocle CellDataSet using the raw count matrix and corresponding cell metadata. Size factors and dispersions were estimated using the estimateSizeFactors() and estimateDispersions() functions, respectively. Ordering genes were selected using differentialGeneTest(), with fibroblast state included as the explanatory variable. Genes with q < 0.01 were retained as candidate ordering genes. To minimize potential technical or stress-related bias, mitochondrial genes, ribosomal genes, and stress-response-associated genes, including Malat1, Xist, Jun, Fos, Egr1, and Atf3, were excluded. When more than 1,500 significant genes were identified, the top 1,500 genes ranked by q-value were used for trajectory construction.

Dimensionality reduction was performed using the DDRTree algorithm, followed by cell ordering with orderCells(). The root state was defined as the Monocle state containing the largest number of Resident Fib cells. The inferred trajectory was interpreted as a putative transcriptional continuum from resident-like fibroblast states toward transitional, ECM-remodeling, and activated secretory fibroblast states. The expression dynamics of Col5a1, Col6a1, Fbn1, and representative fibroblast-state marker genes were visualized along pseudotime. Fibroblast-state module scores were calculated as the average normalized expression of representative marker genes. Pseudotime-dependent genes were identified using differentialGeneTest() with a spline model of pseudotime, and branch-dependent genes were evaluated using BEAM when applicable.

### Ethics statement

The animal experiment in this study was approved by the Experimental Animal Ethics Committee of Zhongnan Hospital of Wuhan University (Ethics approval number: ZN2024004).

### Construction of TAC mouse model

This study was carried out in compliance with the ARRIVE guidelines. 24 male C57BL/6 mice (8-weeks-old) were purchased from Beijing Vital River Laboratory Animal Technology Co., Ltd. The mice were randomly divided into four groups: the sham group, TAC 2 weeks group, TAC 4 weeks group, and TAC 8 weeks group, with six mice in each group. Before surgery, the mice were anesthetized with inhaled isoflurane. Anesthesia was induced with 3% isoflurane in oxygen and maintained with 1.5% isoflurane during surgery. The mice were placed in a supine position on a heating pad to maintain body temperature, and mechanical ventilation was provided after endotracheal intubation. Under sterile conditions, a suprasternal incision was made to expose the transverse aortic arch. A 27-gauge needle was placed alongside the aorta, around which a 6–0 silk suture was tied to induce aortic constriction. The needle was then removed to create a consistent narrowing. The sham group underwent exposure of the aortic arch without ligation. Postoperative care included analgesia and antibiotics. At the designated endpoint, mice were euthanized by cervical dislocation following deep anesthesia induced *via* intraperitoneal injection of sodium pentobarbital at a dose of 40 mg/kg. The euthanasia procedure was performed in accordance with the American Veterinary Medical Association (AVMA) Guidelines for the Euthanasia of Animals: 2020 Edition.

### Real-time quantitative polymerase chain reaction

The expression of key CARGs in TAC mouse model was detected using real-time quantitative polymerase chain reaction (qRT-PCR). Total RNA from cardiac tissue in animal models was extracted using Trizol (Thermo Fisher, USA), and reverse transcription was performed using the Hifair III first Strand cDNA Synthesis SuperMix for qRT-PCR (Ye Sen, China). qRT-PCR was performed using Hieff UNICON Universal Blue qPCR SYBR Green Master Mix (Ye Sen, China) for fluorescent staining of cDNA. GAPDH was used as the reference gene. The relative fold changes of genes were calculated using the 2^−ΔΔCt^ method. The primer sequences used for qPCR are provided in Supplementary Material S5.

### Western blot analysis

Cardiac tissues were homogenized in RIPA lysis buffer (Beyotime, China) containing protease inhibitors (Roche, Switzerland) and lysed on ice for 30 min. The lysates were then centrifuged at 12,000 rpm for 15 min at 4 °C, and the supernatant was collected. Protein concentration was determined using the BCA assay (Beyotime, China). Equal amounts of protein (20 μg) were separated by electrophoresis on a 10% SDS-PAGE gel and subsequently transferred onto PVDF membranes (Millipore, USA). The membranes were blocked with 5% non-fat milk for 1 h at room temperature. The membranes were then incubated overnight at 4 °C with primary antibodies against FBN1 (ABclonal, 1:1000), Col5a1 (ABclonal, 1:1000), COL6A1 (ABclonal, 1:1000), and α-Tubulin (ABclonal, 1:1000). The next day, the membranes were incubated with HRP-conjugated secondary antibodies (Abcam, 1:5000) at room temperature for 1 h. The bands were incubated with ECL chemiluminescent reagent (Beyotime, China) and visualized using the Bio-Rad imaging system (Hercules, USA). Densitometric analysis was performed using ImageJ software, and the results were normalized to α-tubulin levels.

### IHC analysis

Paraffin-embedded mouse hearts were cut transversely into 5-µm sections. After a 5-min high-pressure antigen retrieval process in citrate buffer with a pH of 6.0, the heart sections were blocked with bovine serum albumin for 60 min and then incubated overnight at 4 °C with FBN1, Col5a1, and COL6A1 antibodies. Binding was visualized with the appropriate secondary antibodies for 60 min at 37 °C. Representative images were acquired using a bright-field microscope. The staining intensity/positive staining area was quantified using ImageJ software.

### Statistical analysis

Experimental data are presented as the mean ± standard error of the mean (SEM) after assessment of normal distribution. Data normality was evaluated using the Shapiro-Wilk test, and comparisons between two groups were performed using the t-test. For comparisons among multiple groups in the TAC animal experiments, one-way analysis of variance (ANOVA) was performed, followed by an appropriate *post hoc* multiple-comparisons test where applicable. Pearson correlation analysis was used to assess associations between variables. P < 0.05 was considered statistically significant.

## Results

### Identification and functional enrichment of DEGs

Based on the screening criteria of p value <0.05 and |FoldChange| > 1.5, a total of 836 DEGs were identified in the GSE1621 dataset, including 533 upregulated and 303 downregulated genes (Figure 1A). In the GSE5500 dataset, 1269 DEGs were identified, of which 480 were upregulated and 789 were downregulated (Figure 1B). A total of 143 common DEGs were obtained by intersecting the DEGs from the two datasets (Figure 1C). GO enrichment analysis revealed that these DEGs were mainly enriched in terms such as “cell adhesion”, “biological adhesion”, “extracellular region part”, and “structural molecular activity” (Figure 1D). KEGG enrichment analysis showed that the DEGs were mainly enriched in pathways such as “ECM-receptor interaction”, “Protein digestion and absorption”, and “Focal adhesion” (Figure 1E).

**FIGURE 1:**
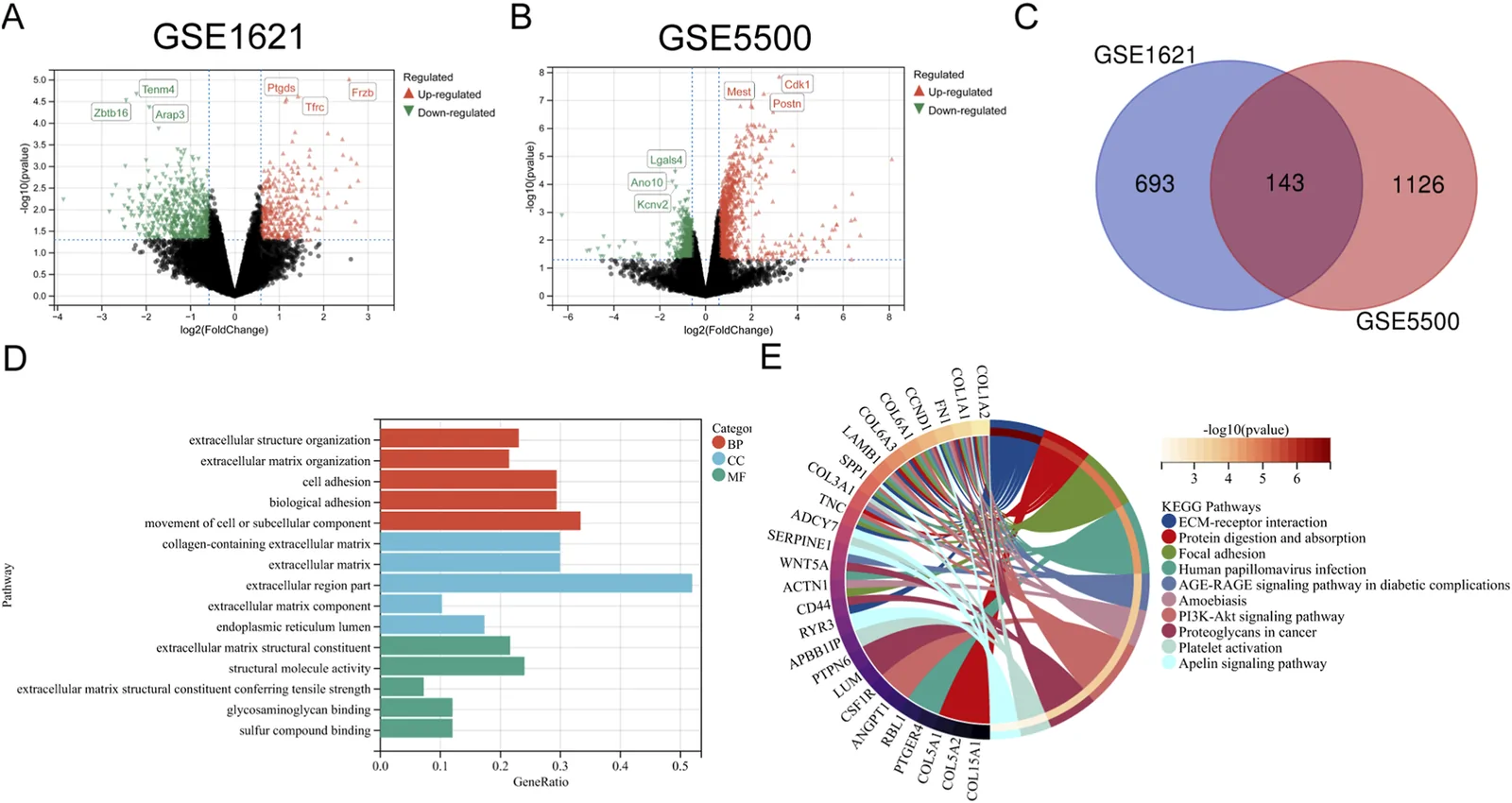
Identification of DEGs and Key Pathways in HF. **(A)** Volcano plot of the GSE1621 dataset. **(B)** Volcano plot of the GSE5500 dataset. **(C)** Venn diagram of common DEGs. **(D)** GO annotation of common DEGs. **(E)** KEGG pathway enrichment analysis of common DEGs.

### Identification and functional enrichment of differential expression CARGs

The intersection of DEGs with CARGs obtained from Msigdb identified 45 differentially expressed CARGs in the HF-related datasets (Figure 2A). GO BP annotation revealed that the differentially expressed CARGs were mainly enriched in terms such as “cell adhesion”, “biological adhesion”, and “cellular development process” (Figure 2B). GO CC annotation showed that these genes were mainly enriched in “extracellular region”, “extracellular region part”, and “extracellular space” (Figure 2C). GO MF annotation indicated that these genes were mainly enriched in “structural molecule activity”, “extracellular matrix structural constituent”, and “signaling receptor binding” (Figure 2D). In the KEGG enrichment analysis, the differentially expressed CARGs were significantly enriched in pathways including “ECM-receptor interaction”, “Focal adhesion”, “Protein digestion and absorption”, “PI3K-Akt signaling pathway”, and “Cell adhesion molecules” (Figure 2E). In the Reactome enrichment analysis, the differentially expressed CARGs were enriched in pathways such as “extracellular matrix organization”, “integrin cell surface interaction”, “degradation of the extracellular matrix”, and “ECM proteoglycans” (Figure 2F).

**FIGURE 2:**
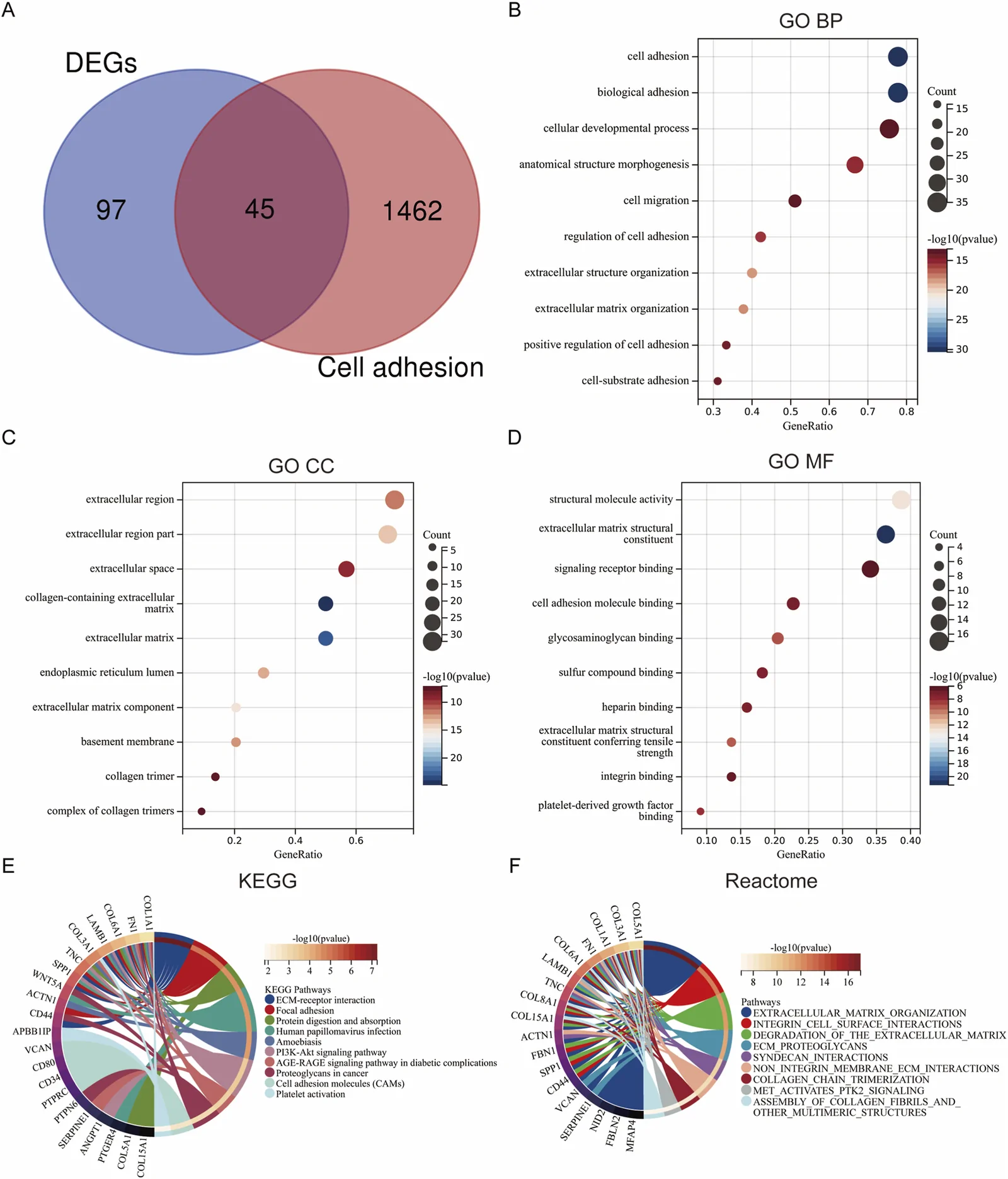
Identification and Functional enrichment of Differential expression CARGs. **(A)** Venn diagram of differential expression CARGs. **(B)** GO BP annotation of differential expression CARGs. **(C)** GO CC annotation of differential expression CARGs. **(D)** GO MF annotation of differential expression CARGs. **(E)** KEGG pathway enrichment analysis of differential expression CARGs. **(F)** Reactome enrichment analysis of differential expression CARGs.

### Construction and validation of the CelladhesionScore

To quantify celladhesion features in individual samples, ssGSEA was performed to calculate the CelladhesionScore for each sample based on the expression levels of the 45 identified differentially expressed CARGs in the training set generated by merging the GSE5500 and GSE1621 datasets. The ssGSEA scores were normalized to a 0–1 range using min-max normalization, and samples were stratified into high- and low-CelladhesionScore groups according to the median CelladhesionScore (Figure 3A). PCA results revealed a certain degree of separation between the high- and low-CelladhesionScore groups, indicating differences in molecular characteristics between the two groups (Figure 3B). The distribution plot indicated that CelladhesionScore varied continuously among different samples (Figure 3C). In the training set, the box plot revealed a significant difference in CelladhesionScore between HF patients and controls (Figure 3D), indicating its ability to discriminate between the two groups. ROC analysis further showed a strong discriminatory ability, with an AUC of 0.988 (P < 0.001) (Figure 3G). The discriminatory ability of CelladhesionScore was further validated in independent HF-related validation sets. In the HF-related mouse dataset GSE18224, CelladhesionScore showed a clear ability to discriminate HF model mice from controls (Figure 3E), and ROC analysis showed an AUC of 0.953 (Figure 3H). In the HF-related human dataset GSE116250, CelladhesionScore similarly demonstrated a clear ability to discriminate HF patients from controls (Figure 3F), with an AUC of 0.846 (P < 0.01) in ROC analysis (Figure 3I).

**FIGURE 3:**
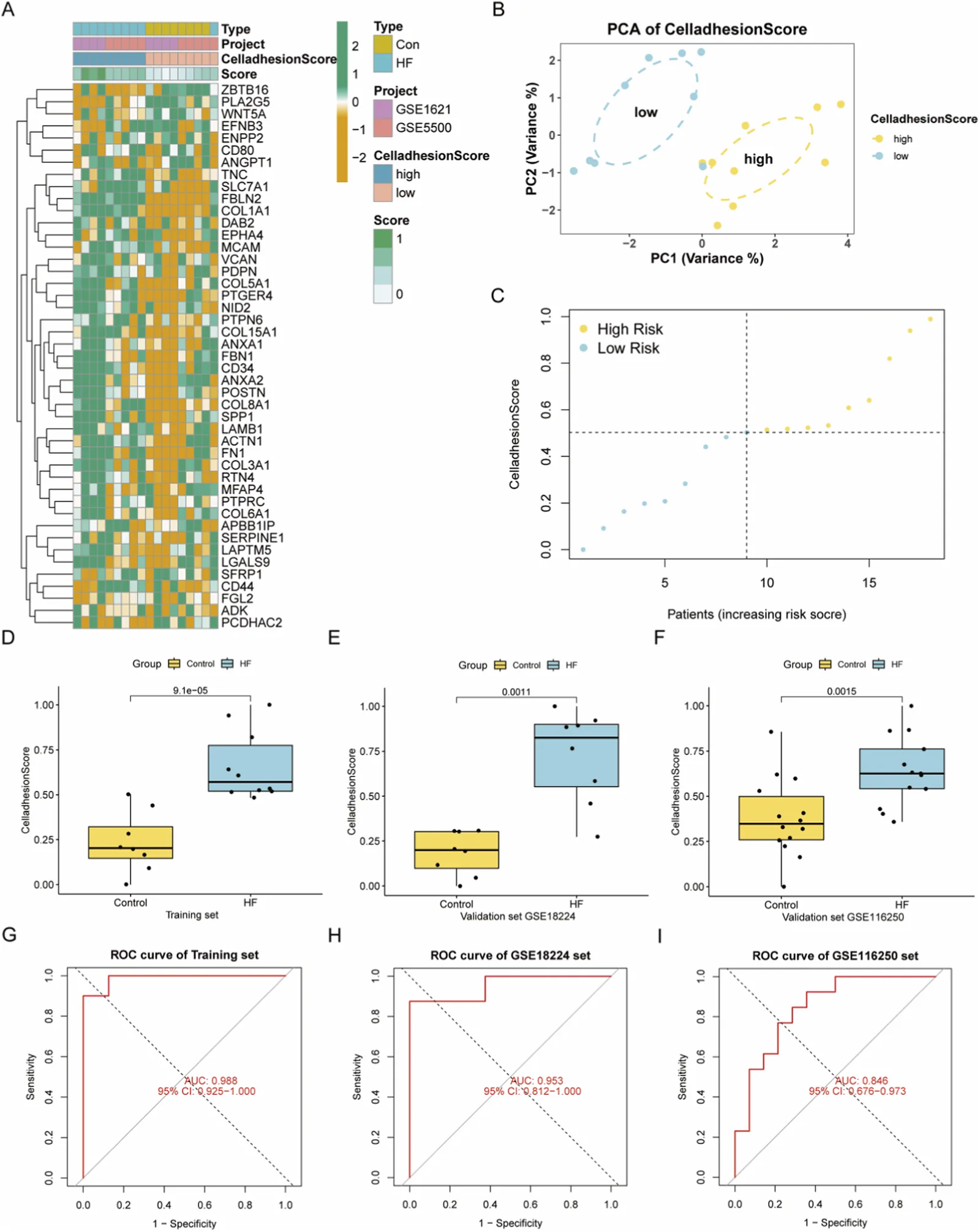
Construction and validation of the CARG-based CelladhesionScore model in HF. **(A)** Heatmap of differentially expressed CARGs and CelladhesionScore-based subtype classification in the training set. **(B)** PCA plot showing the separation between CelladhesionScore-high and CelladhesionScore-low groups. **(C)** Distribution of CelladhesionScore and subtype classification in the training set. **(D)** Comparison of CelladhesionScore between HF and control samples in the training set. **(E)** Validation of CelladhesionScore differences between HF and control samples in GSE18224. **(F)** Validation of CelladhesionScore differences between HF and control samples in GSE116250. **(G)** ROC curve of CelladhesionScore in the training set. **(H)** ROC curve of CelladhesionScore in GSE18224. **(I)** ROC curve of CelladhesionScore in GSE116250.

### Analysis of biological functional differences and fibrosis-related pathways among CelladhesionScore subtypes in HF

GSVA of Hallmark gene sets revealed that several biological processes, including “TGF-β signaling”, “epithelial-mesenchymal transition”, “coagulation”, “angiogenesis”, and “UV response DN”, were enriched in the CelladhesionScore high-risk group (Figure 4A). These findings indicate that a higher CelladhesionScore is associated with broader alterations in remodeling-related signaling programs, among which TGF-β signaling has the most established connection with cardiac fibrosis. Subsequently, the enrichment of fibrosis-related pathways was assessed between the high-risk and low-risk groups. Of the 20 fibrosis-related pathways examined, 11 showed significant differences between the two groups, and all were upregulated in the high-risk group (Figure 4B). Positive correlations were identified between CelladhesionScore and 16 fibrosis-related pathways by Spearman correlation analysis (FDR <0.05). Notably, “Activated Cardiac Fibroblast Expanded” showed the strongest correlation with CelladhesionScore (r = 0.96) (Figure 4C). Among these positively correlated pathways, cardiac fibrosis-related pathways, including “Cardiac Fibroblast Activated” (r = 0.903), “TGFb Smad Fibrosis” (r = 0.879), “CTGF Pericellular Matrix” (r = 0.891), and “Fibronectin Tenascin Network” (r = 0.903), exhibited strong positive correlations with CelladhesionScore (Figures 4D–G).

**FIGURE 4:**
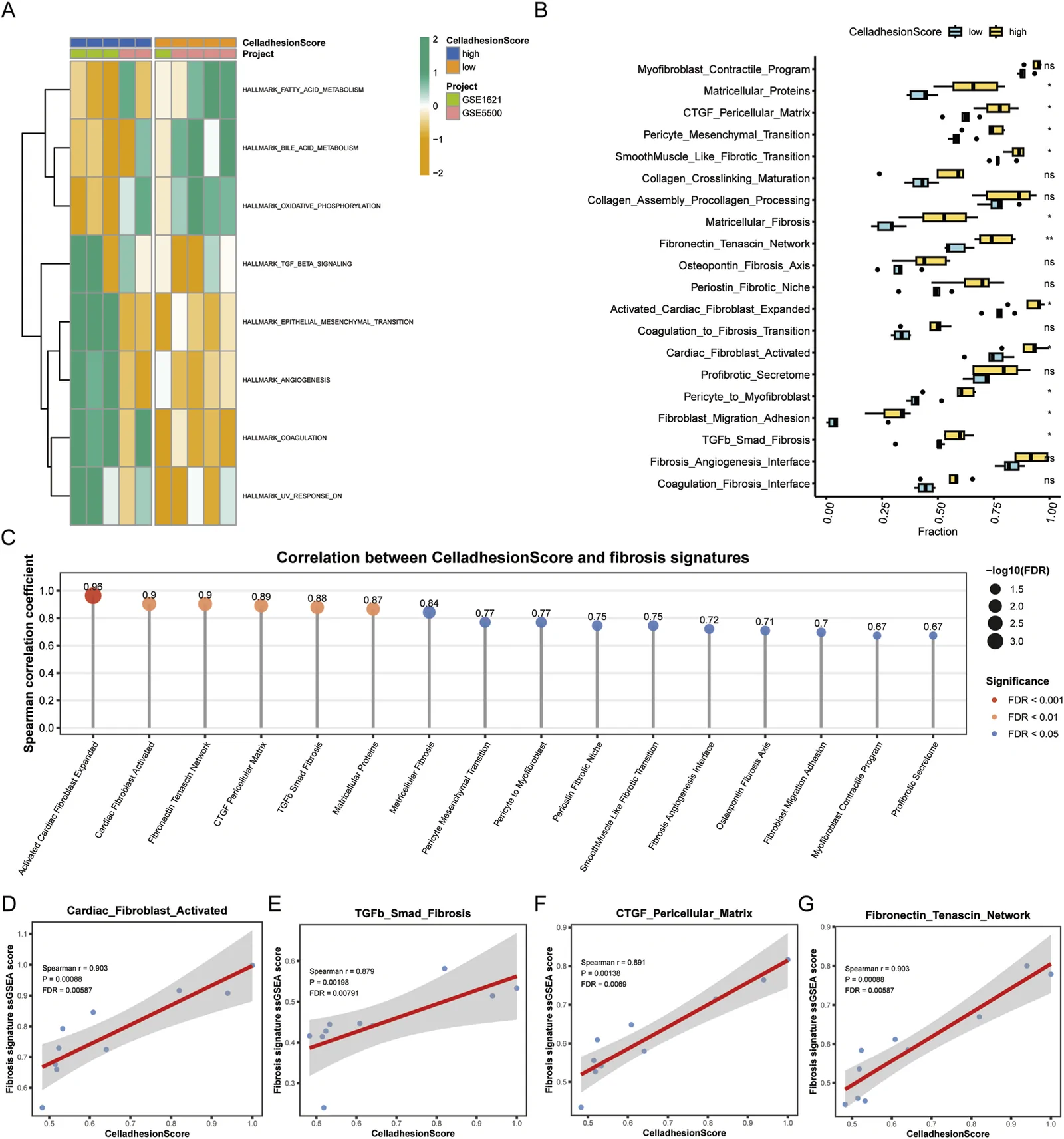
Biological functional differences and fibrosis-related pathway activation between CelladhesionScore subtypes in HF. **(A)** Hallmark pathway enrichment between CelladhesionScore-high and CelladhesionScore-low groups. **(B)** Differential enrichment of fibrosis-related pathways between CelladhesionScore-high and CelladhesionScore-low groups. **(C)** Correlation between CelladhesionScore and fibrosis-related signatures. **(D)** Correlation between CelladhesionScore and Cardiac Fibroblast Activated signature. **(E)** Correlation between CelladhesionScore and TGFβ/Smad Fibrosis signature. **(F)** Correlation between CelladhesionScore and CTGF Pericellular Matrix signature. **(G)** Correlation between CelladhesionScore and Fibronectin–Tenascin Network signature.

### Construction of PPI network and identification of key CARGs

The STRING database was used to perform PPI analysis of the differentially expressed CARGs. The result of the PPI network was visualized using Cytoscape software. After removing unconnected genes, a PPI network was ultimately constructed, consisting of 42 nodes and 183 edges (Figure 5A). Subsequently, hub genes were identified using the DMNC, MCC, and MNC algorithms within the CytoHubba plugin. The 10 hub genes identified by the DMNC algorithm are Angpt1, *Col6a1*, *Nid2*, *Col5a1*, *Lamb1*, *Fbln2*, *Fbn1*, *Col15a1*, *Col8a1*, and *Vcan* (Figure 5B). The 10 hub genes identified by the MCC algorithm are Col15a1, Col8a1, Col1a1, *Col3a1*, *Col6a1*, *Fbln2*, *Fn1*, *Fbn1*, *Postn*, and *Col5a1* (Figure 5C). The 10 hub genes identified by the MNC algorithm are *Postn*, *Cd44*, *Col1a1*, *Col3a1*, *Fn1*, *Ptprc*, *Spp1*, *Col6a1*, *Fbn1*, and *Col5a1* (Figure 5D). By intersecting the hub genes identified through the DMNC, MCC, and MNC algorithms, three key CARGs were ultimately identified: *Col6a1*, *Col5a1*, and *Fbn1* (Figure 5E).

**FIGURE 5:**
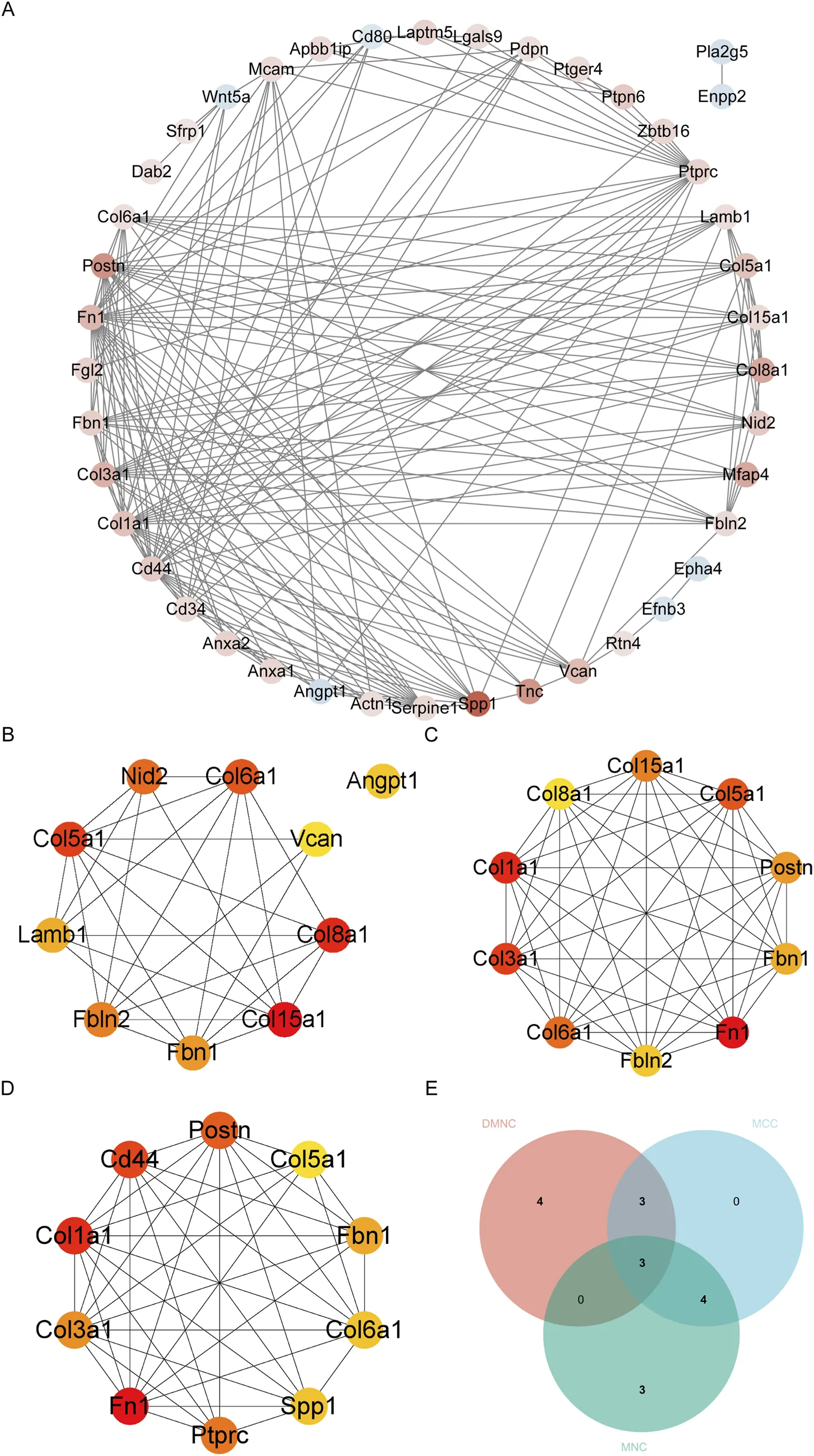
PPI analysis and identification of key CARGs. **(A)** PPI network of differential expression CARGs. **(B)** Identification of hub genes by DMNC algorithm. **(C)** Identification of hub genes by MCC algorithm. **(D)** Identification of hub genes by MNC algorithm. **(E)** Venn diagram of key CARGs by intersecting hub genes.

### Expression patterns of key CARGs in the training and validation datasets

In both the GSE1621 and GSE5500 datasets, *COL6A1*, *Col5a1*, and *FBN1* were highly expressed in the TAC group (Figures 6A,B). In the Pearson correlation analysis, *COL6A1*, *Col5a1*, and *FBN1* exhibited strong correlations with each other in both the GSE1621 and GSE5500 datasets (Figures 6C,D). Finally, in the external dataset GSE36074, *COL6A1*, *Col5a1*, and *FBN1* were also validated to be highly expressed in the TAC group (Figure 6E).

**FIGURE 6:**
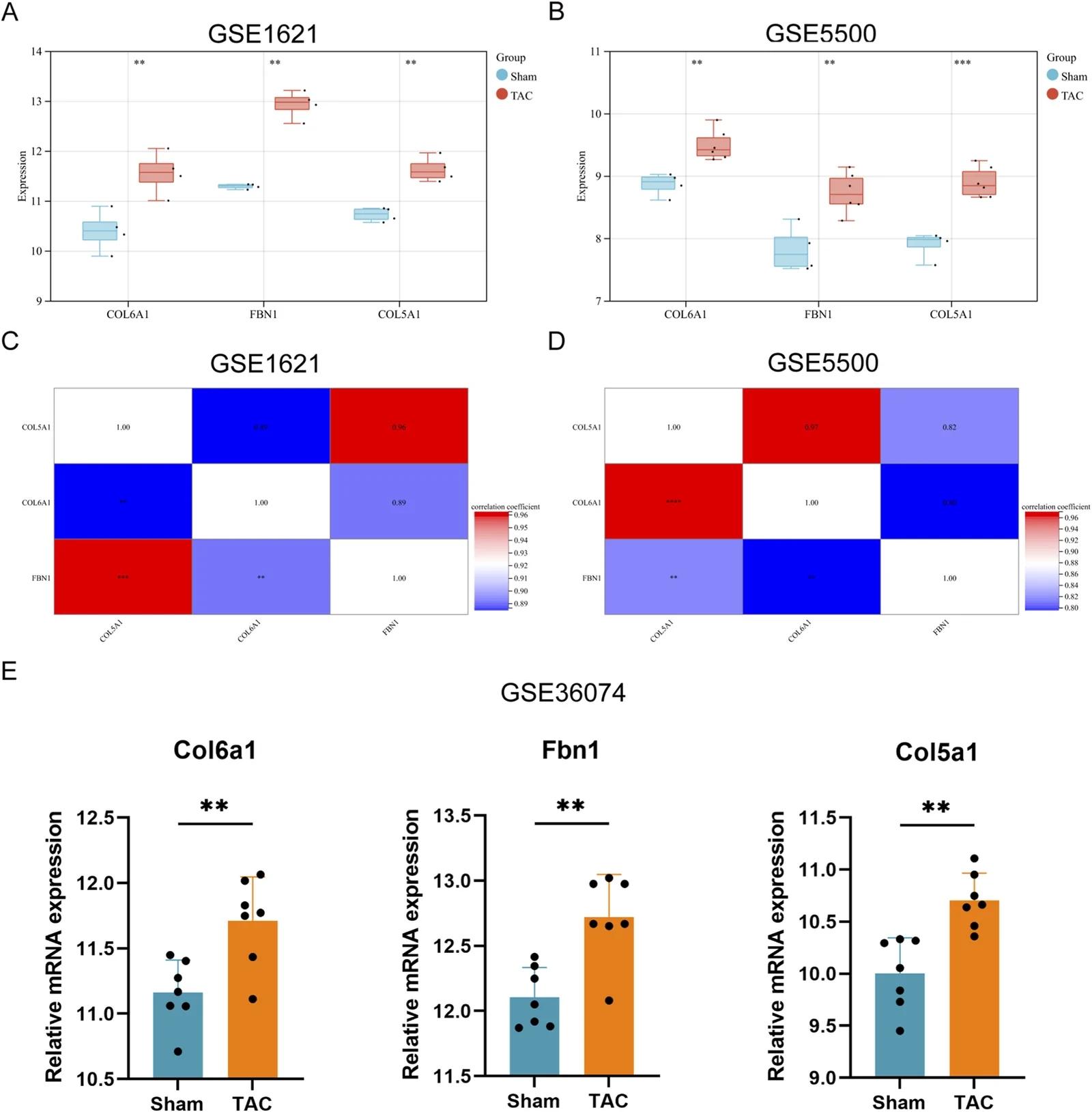
Expression patterns of key CARGs in the training and validation datasets. **(A)** Expression of key CARGs in GSE1621. **(B)** Expression of key CARGs in GSE5500. **(C)** Pearson correlation analysis of key CARGs in GSE1621. **(D)** Pearson correlation analysis of key CARGs in GSE5500. **(E)** Validation of key CARGs in external dataset GSE36074. *P < 0.05; **P < 0.01; ***P < 0.001.

### Single-cell transcriptomic analysis reveals dynamic changes in major cardiac cell populations and key CARGs during TAC progression

To characterize changes in cellular composition during TAC-induced heart failure progression, single-cell transcriptomic analysis was performed on mouse heart samples from the dataset GSE308859, which included one sample for each condition: Sham, TAC 2 weeks, TAC 4 weeks, and TAC 6 weeks. UMAP visualization identified multiple major cardiac cell populations, including cardiomyocytes, fibroblasts, endothelial cells, vascular smooth muscle cells, monocyte/macrophage populations, proliferating immune cells, neutrophils, T cells, NK cells, and B cells (Figure 7A). The expression patterns of canonical marker genes further confirmed the identity of these annotated cell types, supporting the reliability of the cell-type annotation (Figure 7B). UMAP visualization across different TAC time points revealed dynamic changes in the composition of major cell populations during TAC progression. Descriptive comparison across the examined TAC time points suggested apparent differences in the relative abundance of several major cardiac cell populations. Compared with the Sham group, the proportion of monocyte/macrophage populations was mildly increased at TAC 2 weeks, whereas immune cell populations showed an apparent expansion at TAC 4 weeks. At TAC 6 weeks, fibroblasts accounted for a relatively higher proportion, suggesting stage-dependent inflammatory responses and fibrotic remodeling during TAC-induced cardiac remodeling (Figures 7C,D). However, because GSE308859 contains only one biological sample at each time point, these observations should be interpreted as exploratory and descriptive rather than statistically validated temporal changes. Further analysis of the cellular origin of key CARGs revealed that Col5a1, Col6a1, and Fbn1 were predominantly enriched in fibroblasts and displayed dynamic expression patterns during TAC progression (Figure 7E). Expression analysis across different time points further showed that these key CARGs displayed relatively higher expression distributions at TAC 6 weeks, suggesting their potential involvement in late-stage extracellular matrix remodeling and fibrotic progression after TAC (Figure 7F).

**FIGURE 7:**
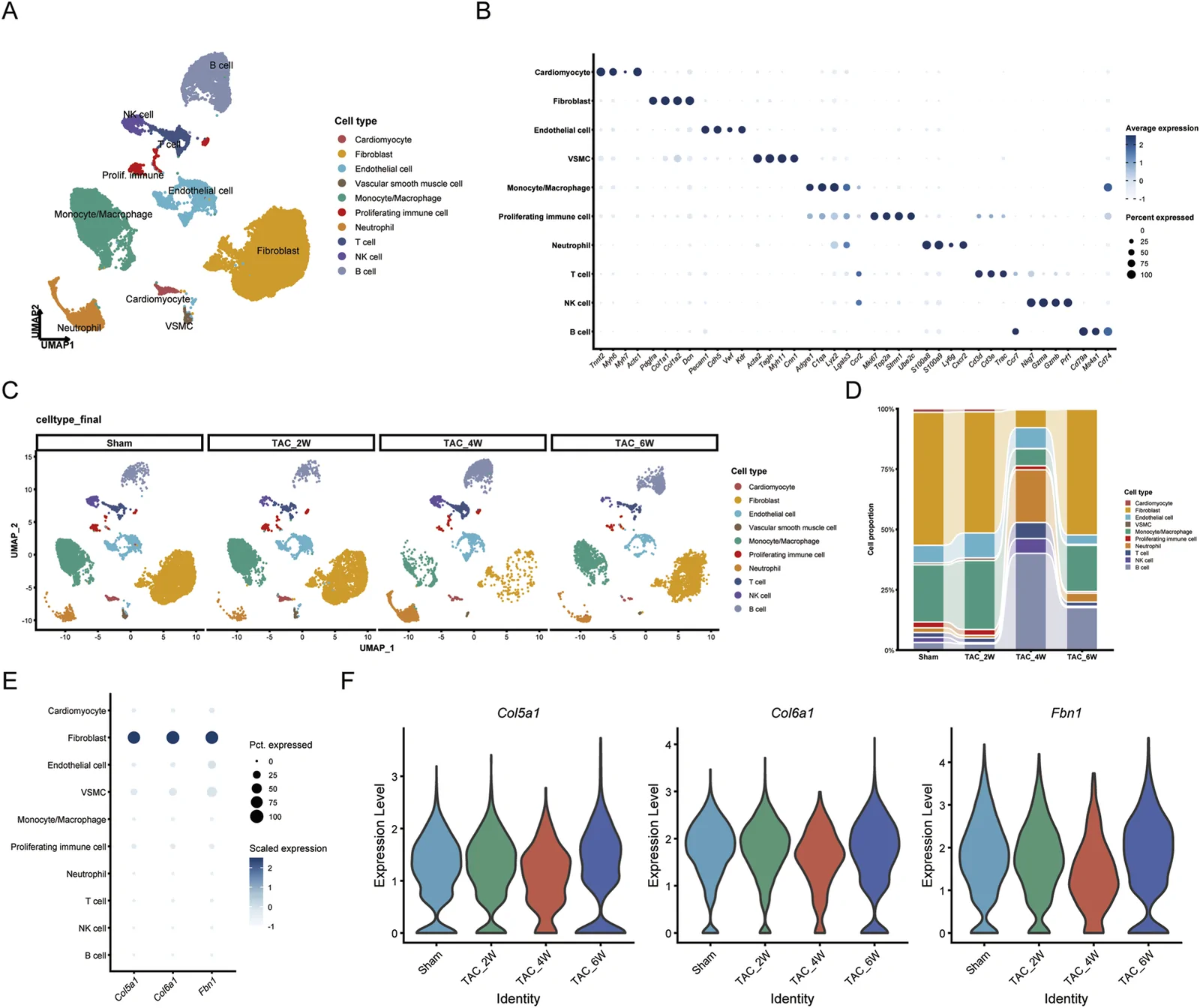
Single-cell transcriptomic analysis reveals dynamic changes in major cardiac cell populations and key CARGs during TAC progression. **(A)** UMAP visualization of major cardiac cell populations. **(B)** UMAP visualization of major cardiac cell populations across TAC progression. **(C)** Dot plot of canonical marker genes for major cardiac cell types. **(D)** Dynamic changes in major cardiac cell proportions across Sham and TAC groups. **(E)** Cellular distribution of key CARGs across major cardiac cell types. **(F)** Expression dynamics of key CARGs across Sham and TAC groups.

### Dynamic changes in fibroblast subpopulation composition during TAC progression

Since key CARGs were predominantly enriched in fibroblasts, fibroblast subpopulation states were further analyzed. Fibroblasts were further stratified into four subpopulations based on representative marker gene expression: Resident Fib, Transitional Fib, ECM-remodeling Fib, and Activated secretory Fib. *Igfbp3*, *Cxcl14*, *Comp*, and *Wif1* were preferentially expressed in these four subpopulations, respectively (Figure 8A). DotPlot further validated the characteristic marker expression of each subpopulation. Resident Fib mainly expressed Igfbp3, Fgl2, Id3, and Apoe; Transitional Fib mainly expressed *Cxcl14*, *Pcsk6*, *Adamts5*, and *Pi16*; ECM-remodeling Fib mainly expressed *Comp*, *Pcolce*, *Lox*, and *Col5a1*; and Activated secretory Fib mainly expressed *Wif1*, *Prg4*, *Clu*, and *Dkk3* (Figure 8B). Fibroblast state proportion analysis showed dynamic changes in different fibroblast states across the Sham, TAC_2W, TAC_4W, and TAC_6W groups, suggesting that fibroblast state transitions accompanied TAC-induced cardiac remodeling (Figures 8C,D).

**FIGURE 8:**
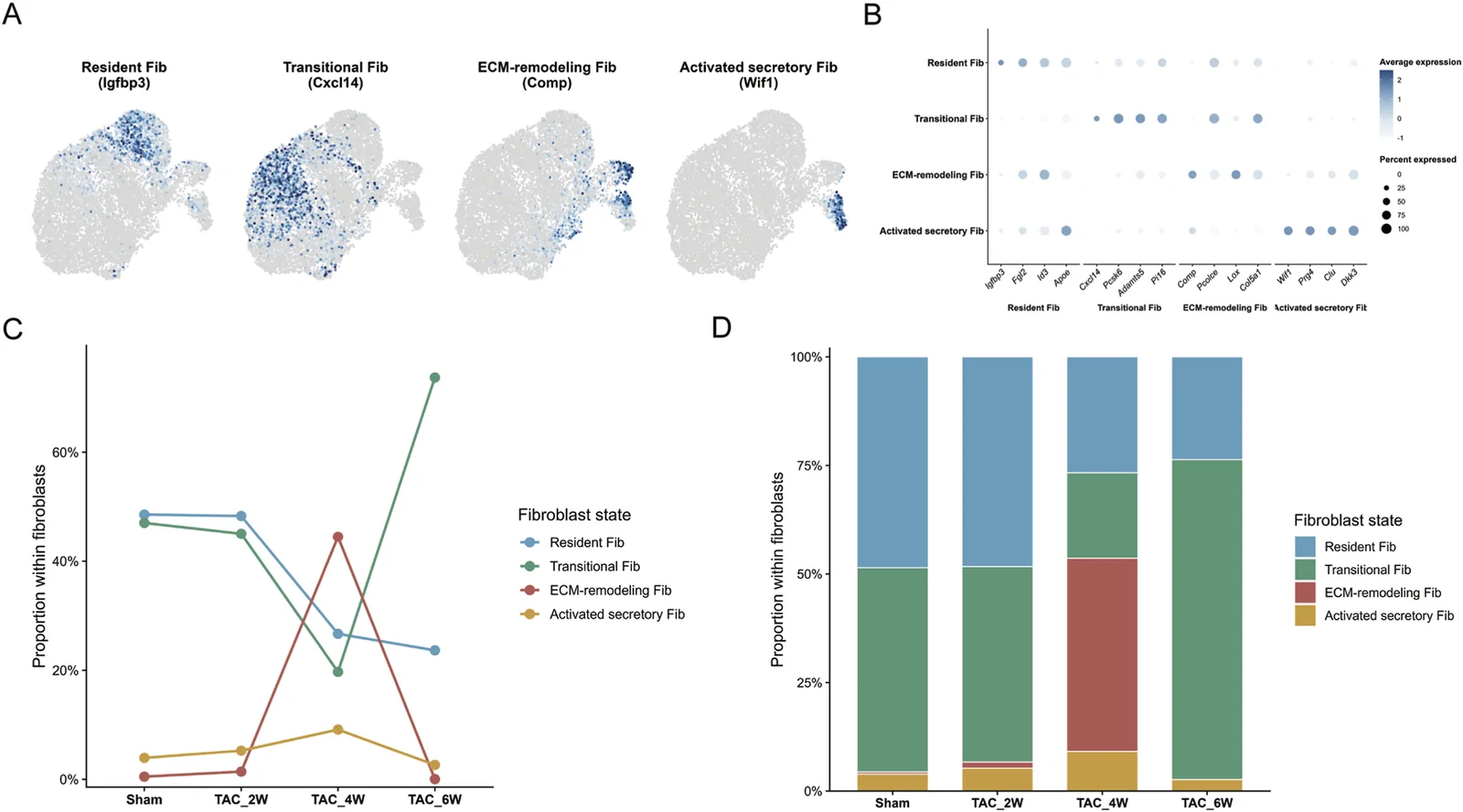
Dynamic changes in fibroblast subpopulation composition during TAC-induced HF progression. **(A)** Feature plots of representative markers for fibroblast subpopulations. **(B)** Dot plot of marker gene expression across fibroblast subpopulations. **(C)** Dynamic changes in fibroblast subpopulation proportions across TAC progression. **(D)** Stacked bar plot showing fibroblast subpopulation composition across Sham and TAC groups.

### Pseudotime analysis reveals fibroblast state transition and dynamic expression of key CARGs during TAC progression

To further investigate fibroblast state transitions and the dynamic expression patterns of CARGs during TAC progression, pseudotime analysis was performed on fibroblasts. Trajectory analysis revealed a branched distribution of different fibroblast states along the pseudotime trajectory. Resident Fib and Transitional Fib were mainly distributed in the early-to-intermediate regions of the trajectory, whereas ECM-remodeling Fib and Activated secretory Fib were predominantly enriched in the late-stage branches, suggesting a potential transition from resident/transitional states toward matrix-remodeling and secretory activated states (Figure 9A). Faceted trajectory plots further demonstrated distinct distributions of fibroblast states along the trajectory. Resident Fib and Transitional Fib occupied the major branches, whereas ECM-remodeling Fib and Activated secretory Fib were mainly enriched in the late-stage upper-right branch, supporting functional diversification during fibroblast state transition (Figure 9B). The composition of different fibroblast states was analyzed along pseudotime. The results showed that Resident Fib and Transitional Fib accounted for the major proportions during early-to-intermediate pseudotime stages, whereas ECM-remodeling Fib and Activated secretory Fib mainly appeared at later pseudotime stages and showed a gradual increase in proportion (Figure 9C). Finally, the pseudotime-dependent expression patterns of key CARGs were analyzed. The results showed that *Col5a1*, *Col6a1*, *and Fbn1* exhibited dynamic expression changes along pseudotime. Among them, *Col5a1* showed a stage-dependent increasing trend in ECM-remodeling Fib, *Col6a1* maintained relatively high expression across multiple fibroblast states, whereas Fbn1 showed a more pronounced increase at later pseudotime stages (Figure 9D).

**FIGURE 9:**
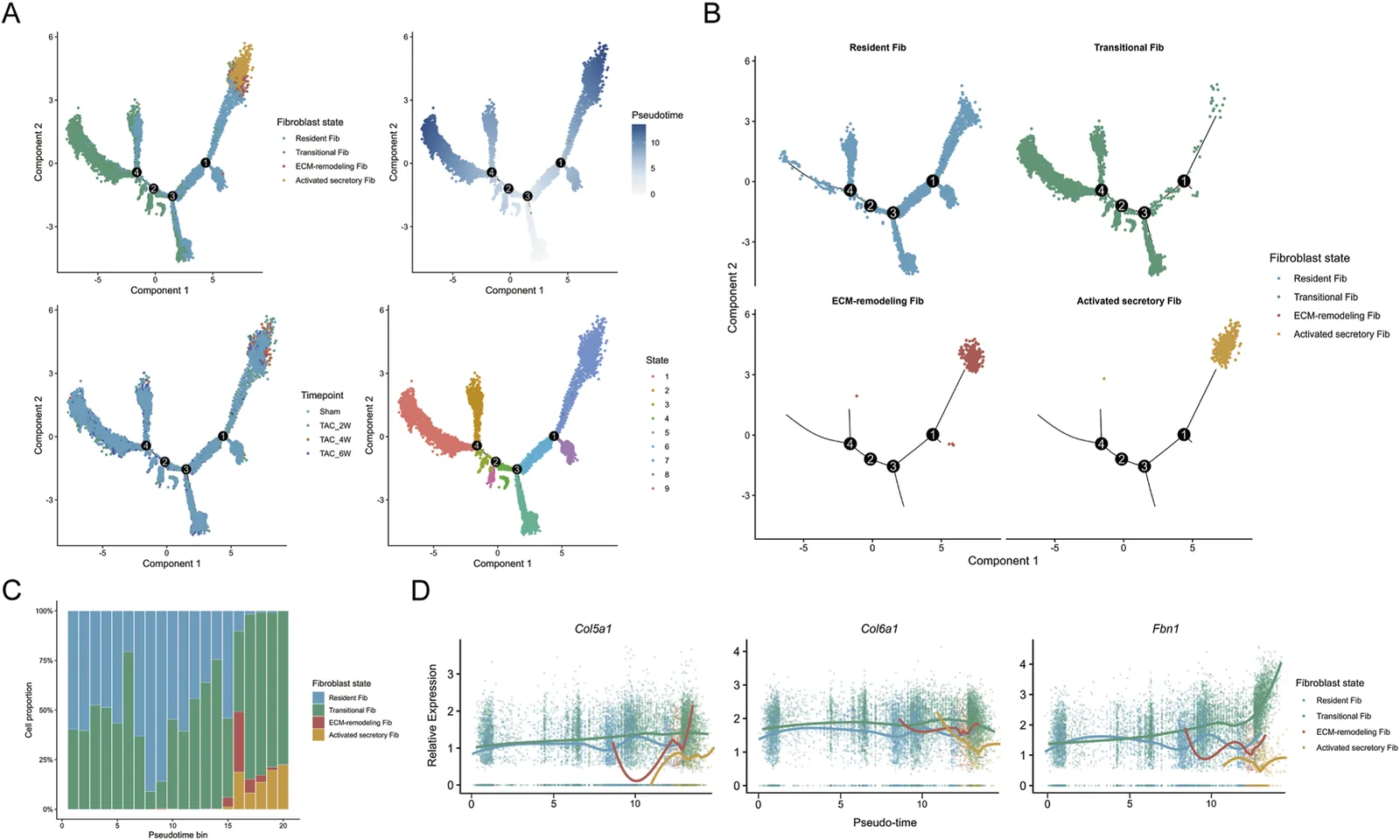
Pseudotime analysis reveals fibroblast state transition and dynamic expression of key CARGs during TAC progression. **(A)** Pseudotime trajectory of fibroblasts during TAC progression. **(B)** Distribution of fibroblast subpopulations along the pseudotime trajectory. **(C)** Changes in fibroblast subpopulation composition along pseudotime. **(D)** Dynamic expression patterns of key CARGs along pseudotime.

### Validation of key CARGs expression in the TAC mouse model

The expression of the three key CARGs was validated in HF mice at different time points after TAC surgery using Western blot, qPCR, and IHC staining. Western blot results showed that the protein levels of *Col5a1*, *Col6a1*, and *Fbn1* progressively increased with the duration of TAC. Notably, differential expression was already evident at 2 weeks post-TAC, with the most pronounced differences observed at 8 weeks (Figure 10A). qPCR results showed that the mRNA expression levels of the three key CARGs were significantly upregulated at 2, 4, and 8 weeks after TAC (Figure 10B). IHC staining revealed that the expression levels of the three key CARGs were progressively upregulated in the cardiac tissues of mice at 2, 4, and 8 weeks after TAC. Compared to the sham group, increased expression of all three genes was observed as early as 2 weeks post-TAC, with further enhancement at 4 and 8 weeks, reaching peak levels at 8 weeks (Figures 11A–C).

**FIGURE 10:**
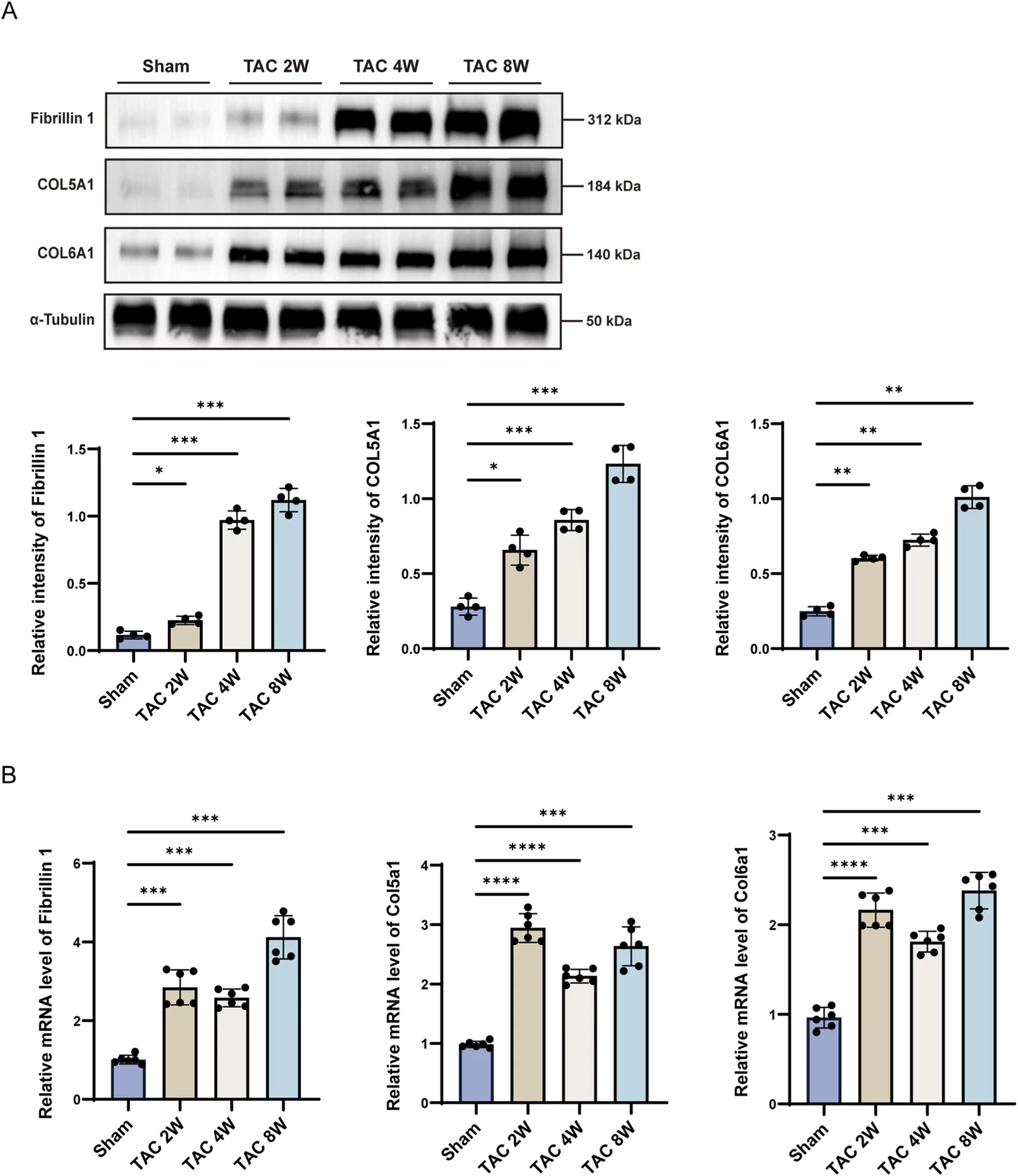
Western blot and qPCR analysis of key CARGs in TAC mouse model. **(A)** Protein expression levels of Fbn1, Col5a1, and Col6a1 in the hearts of Sham mice and mice at 2, 4, and 8 weeks post-TAC. **(B)** mRNA expression levels of Fbn1, Col5a1, and Col6a1 in cardiac tissues of Sham mice and mice at 2, 4, and 8 weeks post-TAC. *P < 0.05; **P < 0.01; ***P < 0.001.

**FIGURE 11:**
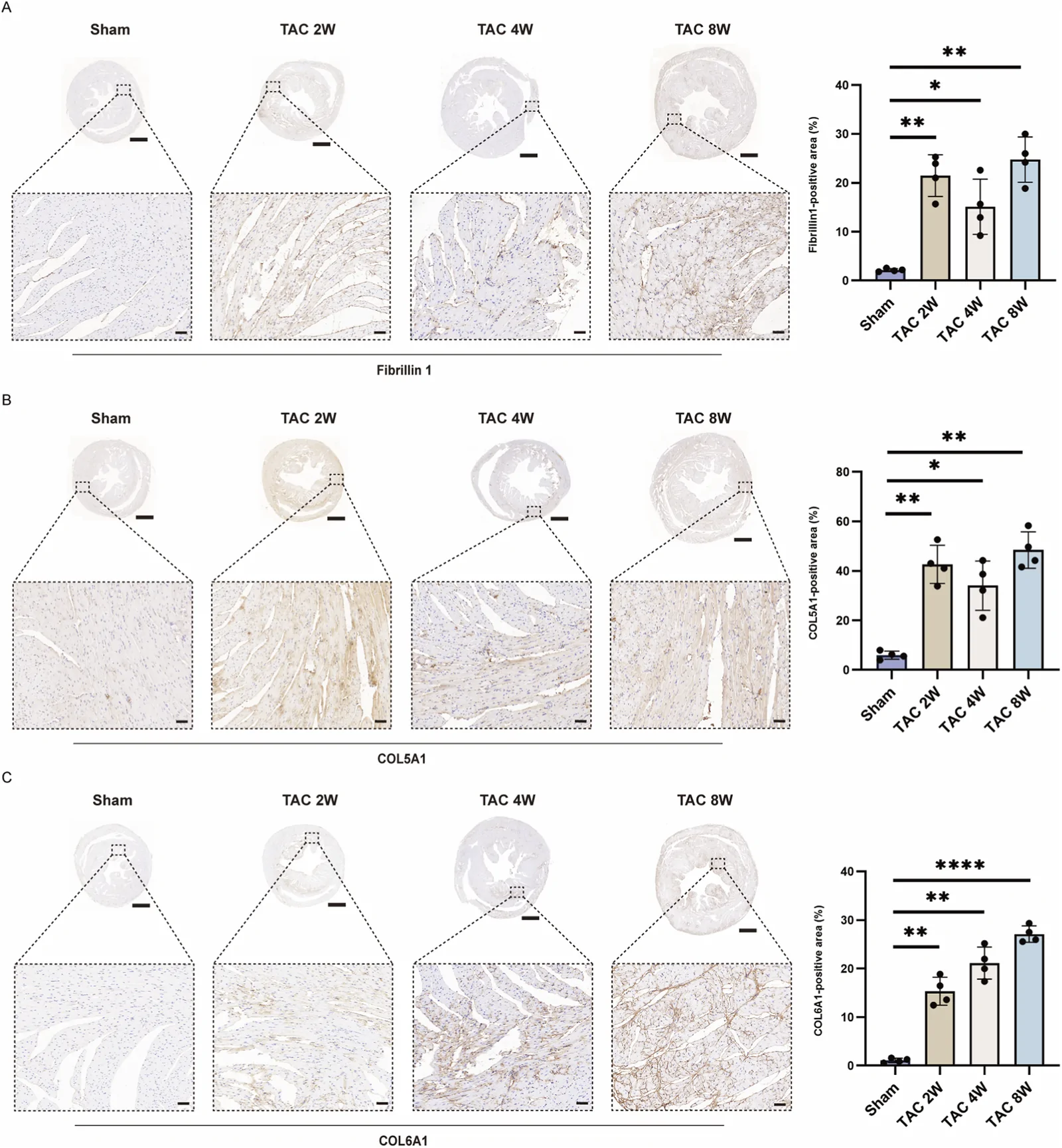
IHC staining of key CARGs in TAC mouse model. **(A–C)** Representative IHC staining images and quantitative analysis of Fbn1, Col5a1, and Col6a1 in cardiac tissue sections from Sham mice and mice at 2, 4, and 8 weeks after TAC. The positive staining area was quantified using ImageJ software. *P < 0.05; **P < 0.01; ***P < 0.001.

## Discussion

The progression of HF is a long-term pathological process, in which maladaptive cardiac remodeling is a major cause leading to the decompensation of heart failure. Myocardial fibrosis is a hallmark of maladaptive cardiac remodeling. Myocardial fibrosis is a hallmark of adverse cardiac remodeling, mainly manifested by the abnormal accumulation of excessive extracellular matrix (ECM) components in myocardial tissue (McKinsey et al., 2023). Under pathological stimuli of HF, cardiac fibroblasts are activated into myofibroblasts, which synthesize and secrete large amounts of collagen and other ECM components. This leads to myocardial interstitial proliferation and increased myocardial stiffness, ultimately resulting in reduced cardiac compliance and impaired pumping function (Segura et al., 2014). The key to the interaction between cells and the ECM lies in cell adhesion molecules. Cell adhesion molecules, such as integrins, mediate cell attachment to the ECM, thereby preserving the structural integrity of organs (Kanchanawong and Calderwood, 2023). Meanwhile, intercellular adhesion molecules are also involved in a variety of signaling pathways directly connecting cells to the extracellular matrix (ECM), such as the PI3K/Akt and MAPK pathways, thereby mediating cellular processes including proliferation, migration, and apoptosis (Romani et al., 2021; Chastney et al., 2025). Moreover, cell adhesion molecules such as ICAM and VCAM can mediate the adhesion, rolling, and transmigration of inflammatory cells across vascular endothelial cells, thereby facilitating their recruitment to sites of inflammation (Filippi, 2019). These infiltrating inflammatory cells secrete pro-inflammatory cytokines within the heart, which in turn activate fibroblasts to produce extracellular matrix components such as collagen, thereby promoting myocardial fibrosis (Prabhu and Frangogiannis, 2016).

In this study, we identified 143 common DEGs from two HF-related transcriptomic datasets. Enrichment analysis indicated that these DEGs were mainly involved in biological processes such as “cell adhesion”, “biological adhesion”, “extracellular region part”, “structural molecular activity”, and “ECM-receptor interaction”. This highlights the critical role of cell-ECM adhesion and ECM-related fibrosis in the pathogenesis of HF. Subsequently, we identified 45 differentially expressed CARGs, all of which were enriched in pathways related to cell adhesion and ECM components. To further characterize cell adhesion-related molecular alterations at the individual-sample level, we constructed a CelladhesionScore model based on the 45 differentially expressed CARGs. This score effectively distinguished HF samples from control samples in the training set and maintained favorable diagnostic performance in two independent validation datasets, suggesting that dysregulated cell adhesion-related signatures may represent stable molecular features of HF. Notably, this model maintained good discriminatory ability in both human and mouse HF-related datasets, suggesting that the expression alterations of these CARGs may represent stable and conserved molecular features during HF progression. Enrichment analysis further revealed the biological significance of the CelladhesionScore. The CelladhesionScore-high risk group was enriched in several fibrosis and remodeling-related pathways including “TGF-β signaling”, “epithelial–mesenchymal transition”, “coagulation”, and “angiogenesis”. Among these pathways, TGF-β signaling and epithelial-mesenchymal transition are closely associated with fibroblast activation (Shu et al., 2022), extracellular matrix deposition (Zeisberg et al., 2007), and cardiac remodeling (Rosenkranz, 2004). Among these differentially expressed CARGs, three key genes (*Col6a1*, *Col5a1*, and *Fbn1*) were further identified using multiple analytical algorithms. These three key CARGs were consistently upregulated in the HF groups of the training datasets, external validation dataset, and in the mouse TAC model. Collagen type V is a minor fibrillar collagen in ECM. Although it is present in low abundance, it plays a crucial role in regulating collagen fibrillogenesis, maintaining tissue structural integrity, and mediating cell-ECM interactions. Col5a1 encodes the α1 chain of collagen type V, which is present at relatively low abundance in the extracellular matrix. Rather than serving merely as a structural filler, type V collagen functions as a regulatory fibrillar collagen that co-assembles with type I collagen to form heterotypic collagen fibrils and regulates fibril nucleation, diameter, and organization, thereby playing an important role in maintaining the structural integrity and mechanical properties of the extracellular matrix (Mak et al., 2007). Collagen VI is involved in the formation of microfibrils and is primarily composed of α1, α2, and α3 chains. It plays a broad role in mediating cell-ECM interactions and signal transduction. Col6a1 encodes the α1 chain of collagen type VI. A previous study has suggested that type VI collagen is not merely a structural component but can also regulate fibroblast-to-myofibroblast differentiation, cell adhesion, and matrix contraction, thereby exerting regulatory effects on signaling processes during fibrosis (Williams et al., 2020). Fbn1 encodes fibrillin-1, a structural component of load-bearing microfibrils in non-collagenous connective tissues, primarily associated with the integrin signaling pathway. Fbn1 is a key pathogenic gene in Marfan syndrome; its mutations can lead to impaired elastic fibers, disruption of ECM homeostasis, and reduced mechanical properties of connective tissue, ultimately resulting in the development of Marfan syndrome (Pinard et al., 2019).

These key CARGs also have translational potential that warrants further investigation. A previous study have shown that type V collagen, encoded by Col5a1, can regulate scar size after cardiac injury. Loss of type V collagen has been reported to exacerbate scar formation and cardiac dysfunction following ischemic cardiac injury and to enhance integrin-dependent fibroblast activation (Yokota et al., 2020). This finding suggest that *Col5a1* is not merely a structural component of the extracellular matrix but may also participate in matrix-fibroblast activation feedback during cardiac remodeling. Similarly, a previous study reported that *Col6a1* deficiency resulted in reduced collagen deposition, a smaller infarct size, and better preserved cardiac function in mice following myocardial infarction (Luther et al., 2012). As a key component of extracellular microfibrils, Fbn1 is markedly upregulated during myocardial fibrosis, and Ang II and TGF-β1 can directly promote Fbn1 synthesis in cardiac fibroblasts (Bouzeghrane et al., 2005), suggesting a close association with fibroblast activation and extracellular matrix remodeling. Further studies have shown that mice with Fbn1 dysfunction are more susceptible to cardiac decompensation, myocardial fibrosis, and ventricular dilation under pressure overload, whereas inhibition of TGF-β, AT1R, or ERK signaling can attenuate these pathological phenotypes (Rouf et al., 2017). In summary, these key CARGs may represent promising candidate targets for therapeutic intervention in cardiac fibrosis and adverse cardiac remodeling. Beyond these key CARGs, several other extracellular matrix- and adhesion-related molecules have also shown translational potential in heart failure. Circulating collagen turnover markers, including procollagen type I C-terminal propeptide (*PICP*) and procollagen type III N-terminal propeptide (*PIIINP*), have been associated with myocardial fibrosis, adverse remodeling, and clinical outcomes in patients with heart failure (Ravassa et al., 2023). Galectin-3 has also been investigated as a circulating biomarker reflecting fibroblast activation and extracellular matrix remodeling (de Boer et al., 2010). These findings further support the translational relevance of adhesion- and matrix-related pathways in the diagnosis, risk stratification, and therapeutic targeting of cardiac fibrosis and heart failure.

Single-cell transcriptomic analysis further demonstrated the association between the key CARGs and HF progression. In the single-cell transcriptomic results, we found that the proportions of major cardiac cell populations changed dynamically during TAC-induced cardiac remodeling. At TAC 2 weeks, monocyte/macrophage populations were increased, whereas immune cell populations were expanded at TAC 4 weeks. By TAC 6 weeks, fibroblasts accounted for a relatively higher proportion. These dynamic changes suggest that TAC-induced HF may involve a stage-dependent transition from inflammatory responses to fibrotic remodeling. Importantly, our analysis showed that *Col5a1*, *Col6a1*, and *Fbn1* were predominantly enriched in fibroblasts and exhibited relatively higher expression at TAC 6 weeks, suggesting that these key CARGs may be closely associated with late-stage extracellular matrix remodeling and fibrotic progression in HF.

Since the key CARGs were predominantly enriched in fibroblasts, we further analyzed fibroblast heterogeneity during TAC progression and identified four fibroblast subpopulations: Resident Fib, Transitional Fib, ECM-remodeling Fib, and Activated secretory Fib. Resident Fib was characterized by the preferential expression of *Igfbp3*, *Fgl2*, *Id3*, and *Apoe*, which are involved in growth factor regulation (Wang et al., 1996), immunomodulation (Liu et al., 2008), cell differentiation (Zebedee and Hara, 2001), and lipid metabolism (Zhang et al., 2011), respectively. These molecular features suggest that Resident Fib may represent a relatively homeostatic fibroblast state, primarily contributing to the maintenance of myocardial tissue architecture and extracellular matrix homeostasis. Transitional Fib was characterized by high expression of *Cxcl14*, *Pcsk6*, *Adamts5*, and *Pi16*. Among these markers, Cxcl14 is a chemokine involved in immune cell recruitment during inflammatory responses (Lu et al., 2016), *Adamts5* mainly participates in the degradation of extracellular matrix components (Barallobre-Barreiro et al., 2021), and *Pcsk6* is involved in extracellular precursor protein processing and is associated with proteolytic networks related to ECM remodeling (Kuhn et al., 2020). These molecular features suggest that this fibroblast subpopulation may represent an intermediate state linking inflammatory signaling with extracellular matrix turnover. ECM-remodeling Fib was characterized by the representative markers *Comp* (Halász et al., 2007), *Pcolce* (Bourhis et al., 2013), *Lox* (Lópe et al., 2010), and *Col5a1* (Wenstrup et al., 2004), which are closely involved in collagen processing, matrix organization, and extracellular matrix remodeling.Notably, *Col5a1* was not only identified as a key CARG in our study, but also served as a representative marker of ECM-remodeling Fib. This finding further links the cell adhesion-related gene signature with fibroblast-mediated ECM remodeling. Activated secretory Fib was characterized by the representative markers *Wif1*, *Prg4*, *Clu*, and *Dkk3*. *Wif1* and *Dkk3* are secreted regulatory factors involved in Wnt signaling modulation (Hsieh et al., 1999; Ni and ehrs, 2006), whereas Clu encodes a secreted molecular chaperone associated with cellular stress responses and tissue remodeling (Wilson and Easterbrook-Smith, 2000). Prg4 also encodes a secreted protein (Rhee et al., 2005). These molecular features suggest that Activated secretory Fib may represent a fibroblast state associated with enhanced secretory activity and signaling regulation.

Pseudotime analysis further demonstrated that fibroblasts undergo dynamic changes during HF progression. Among the fibroblast subpopulations, Resident Fib and Transitional Fib were mainly distributed in the early-to-intermediate regions of the pseudotime trajectory, whereas ECM-remodeling Fib and Activated secretory Fib were predominantly enriched in late-stage branches. This pattern suggests a potential transition of fibroblasts from homeostatic or transitional states toward matrix-remodeling and secretory activated phenotypes. In addition, the key CARGs also exhibited dynamic expression patterns along the pseudotime trajectory. *Col5a1* exhibited a progressive increase in ECM-remodeling Fib, while *Fbn1* showed increased expression in late-stage Resident Fib. By contrast, *Col6a1* maintained relatively stable expression across multiple fibroblast states. Given that ECM-remodeling Fib was characterized by high *Col5a1* expression, together with the observed dynamic changes along pseudotime, these findings suggest that *Col5a1* may participate in matrix remodeling and extracellular matrix deposition during HF progression. As an important component of extracellular microfibrils, the increased expression of Fbn1 in late-stage Resident Fib suggests that this resident-like fibroblast population may contribute to extracellular matrix maintenance during late-stage HF progression. The relatively stable expression of *Col6a1* across fibroblast states suggests that it may exert a broader function in fibroblast-mediated matrix remodeling and ECM homeostasis, rather than being specifically associated with a particular activation state.

This study has several limitations. In this study, the initial screening of differentially expressed CARGs was mainly based on microarray transcriptomic datasets derived from early time points after TAC surgery. Compared with bulk RNA-seq, microarray platforms have certain limitations in transcriptome coverage and dynamic detection range. In addition, early-stage TAC samples may preferentially reflect acute or compensatory cardiac remodeling responses and may not fully represent the molecular features of late-stage or end-stage heart failure. Although the key CARGs and the CelladhesionScore were further evaluated in independent datasets and in a TAC mouse model at multiple time points, the potential bias introduced by the initial dataset selection cannot be completely excluded. Future studies based on bulk RNA-seq data covering multiple stages of TAC-induced cardiac remodeling, as well as longitudinal human HF cohorts, are needed to further validate and refine the applicability of the CelladhesionScore. The CelladhesionScore was constructed based on an integrated dataset with a relatively limited sample size. In addition, because the CelladhesionScore was initially constructed using only two microarray-based discovery datasets, GSE1621 and GSE5500, the limited number of datasets and platform-specific characteristics may introduce dataset-specific bias and raise concerns about potential overfitting. Therefore, its generalizability to broader HF populations, other transcriptomic platforms, and different stages of HF progression remains to be further validated. Although the CelladhesionScore was calculated using the established ssGSEA method, we did not systematically compare its performance with other gene signature scoring approaches, such as UCell, AUCell, GSVA, or z-score-based methods. Therefore, the potential influence of different scoring algorithms on the robustness and generalizability of the CelladhesionScore cannot be fully excluded. Future studies comparing multiple scoring strategies across microarray, bulk RNA-seq, and single-cell RNA-seq datasets are needed to further evaluate and optimize the applicability of the CelladhesionScore. Although its ability to discriminate HF samples from controls was validated in independent datasets, prospective validation in large-scale human HF cohorts is still required. In addition, although changes in the expression of the key CARGs were validated in a TAC mouse model, their roles in fibroblast activation, ECM deposition, and cardiac dysfunction following HF remain unclear. Further *in vitro* and *in vivo* gain- and loss-of-function experiments are therefore needed to elucidate their molecular and biological functions. The single-cell transcriptomic analysis in this study was based on the GSE308859 dataset, which contains only one biological sample at each examined time point. Therefore, the single-cell transcriptomic results cannot be interpreted as statistically validated temporal dynamic changes during TAC progression. Further single-cell studies with sufficient biological replicates and lineage-tracing experiments are still needed to validate these findings. Moreover, the Supplementary Material further explored hypotheses regarding the potential immune associations, upstream miRNA regulation, and candidate compound interactions of the key CARGs. These findings were derived from transcriptomic correlation analyses, database-based predictions, and *in silico* molecular docking, respectively, and therefore require validation in independent experimental studies.

## Conclusion

This study identified *Col5a1*, *Col6a1*, and Fbn1 as key CARGs in HF. Microarray-based and single-cell transcriptomic analyses indicated that these genes were primarily associated with fibroblast-mediated ECM remodeling and exhibited distinct expression patterns across different fibroblast states. Their upregulation in HF was further confirmed in independent datasets and in a TAC mouse model. These findings provide a new framework for understanding the relationships among cell adhesion, fibroblast function, and HF, and suggest that these genes may represent potential molecular candidates for therapeutic intervention in HF.

## Data Availability

The datasets presented in this study can be found in online repositories. The names of the repository/repositories and accession number(s) can be found below: https://www.ncbi.nlm.nih.gov/geo/query/acc.cgi?acc=GSE1621, https://www.ncbi.nlm.nih.gov/geo/query/acc.cgi?acc=GSE5500, https://www.ncbi.nlm.nih.gov/geo/query/acc.cgi?acc=GSE36074, https://www.ncbi.nlm.nih.gov/geo/query/acc.cgi?acc=GSE18224, https://www.ncbi.nlm.nih.gov/geo/query/acc.cgi?acc=GSE308859, https://www.ncbi.nlm.nih.gov/geo/query/acc.cgi?acc=GSE116250.
