# Supplementary material for: Integrated microarray-based and single-cell transcriptomic analyses reveal cell adhesion-related fibrotic remodeling signatures in heart failure
Source: Front Mol Biosci. 2026 Sep 22;13:1929610. doi: 10.3389/fmolb.2026.1929610 (PMC13640610; doi:10.3389/fmolb.2026.1929610)
Supplement: Supplementary file 1 [file DataSheet1.docx]

**Figure S1 Sensitivity analysis of the association between CelladhesionScore and fibrosis signatures after removal of overlapping genes**

**Figure S2 Independent Slingshot trajectory inference and post hoc mapping of fibroblast states**

**Figure S3 Phenotypic validation of the TAC model and fibroblast-associated localization of key CARGs**
