# Supplementary material for: Integrated microarray-based and single-cell transcriptomic analyses reveal cell adhesion-related fibrotic remodeling signatures in heart failure
Source: Front Mol Biosci. 2026 Sep 22;13:1929610. doi: 10.3389/fmolb.2026.1929610 (PMC13640610; doi:10.3389/fmolb.2026.1929610)
Supplement: Supplementary file 2 [file Supplementaryfile1.docx]

**Method**

***Identification of the Immune Infiltration Landscape in the key CARGs***

The immune features of key CARGs, including immune cell infiltration and immune response activity, in the GSE1621 and GSE5500 datasets were evaluated using single-sample gene set enrichment analysis (ssGSEA). The immune signatures, including 23 immune cell infiltration profiles and 17 immune response activity patterns, were acquired from the Immunology Database and Analysis Portal database (https://www.immport.org/home).

**Result**

***Identification of the Immune Infiltration Landscape in the key CARGs***

Immune cell infiltration analysis revealed that in both the GSE1621 and GSE5500 datasets, the three key CARGs were strongly positively correlated with “Natural killer cell”, “Activated CD4 T cell”, “Natural killer T cell”, and “Activated dendritic cell”, while showing a strong negative correlation with “CD56bright natural killer cell” (Figure S1 1 A and C). In the immune response infiltration analysis, the three key CARGs showed strong positive correlations with “TGFβ Family Member” and “Antigen Processing and Presentation” in both datasets, while they were negatively correlated with “Interferons”, “TGFβ Family Member Receptor”, “TNF Family Member”, and “Interferon Receptor” (Figure S1 B and D).

**Figure S1. Identification of the Immune Infiltration Landscape in the key CARGs**

(A) Immune cell infiltration analysis of key CARGs in GSE1621. (B) Immune response infiltration analysis of key CARGs in GSE1621. (C) Immune cell infiltration analysis of key CARGs in GSE5500. (D) Immune response infiltration analysis of key CARGs in GSE5500.
