# Supplementary material for: Integrated microarray-based and single-cell transcriptomic analyses reveal cell adhesion-related fibrotic remodeling signatures in heart failure
Source: Front Mol Biosci. 2026 Sep 22;13:1929610. doi: 10.3389/fmolb.2026.1929610 (PMC13640610; doi:10.3389/fmolb.2026.1929610)
Supplement: Supplementary file 3 [file Supplementaryfile2.docx]

**Method**

***Prediction of miRNAs targeting the key CARGs***

To explore potential regulatory mechanisms of the key CARGs, online databases were used to predict miRNAs targeting these genes. miRNA predictions were performed using TargetScan ([https://www.targetscan.org/vert_80/](https://www.targetscan.org/vert_80/)), miRDB ([https://mirdb.org/](https://mirdb.org/)), and miRWalk ([http://mirwalk.umm.uni-heidelberg.de/](http://mirwalk.umm.uni-heidelberg.de/)), and the intersecting results from these three databases were identified as the miRNAs targeting the key CARGs.

**Result**

***Prediction of miRNAs targeting the key CARGs***

According to the combined predictions from three online databases (TargetScan, miRDB, and miRWalk), 80 miRNAs were predicted to target Col5a1 (Figure S2 A), 34 miRNAs to target Col6a1 (Figure S2 B), and 110 miRNAs to target Fbn1 (Figure S2 C). Among all the predicted miRNAs, hsa-miR-125b-2-3p, hsa-miR-29b-3p, hsa-miR-25-3p, hsa-miR-92a-3p, hsa-miR-92b-3p, hsa-miR-676-5p, hsa-miR-29c-3p, and hsa-miR-3148 were found to target both Col5a1 and Fbn1. Additionally, hsa-miR-6883-5p was predicted to target both Col5a1 and Col6a1, while hsa-miR-5682 and hsa-miR-6871-3p were predicted to simultaneously target Col5a1, Col6a1, and Fbn1 (Figure S2 D).

**Figure S2. Prediction of miRNAs targeting the key CARGs**

(A) miRNA-mRNA network of Col5a1. (B) miRNA-mRNA network of Cola6a1. (C) miRNA-mRNA network of Fbn1. (D) miRNAs co-targeting key CARGs.
