# Supplementary material for: Integrated microarray-based and single-cell transcriptomic analyses reveal cell adhesion-related fibrotic remodeling signatures in heart failure
Source: Front Mol Biosci. 2026 Sep 22;13:1929610. doi: 10.3389/fmolb.2026.1929610 (PMC13640610; doi:10.3389/fmolb.2026.1929610)
Supplement: Supplementary file 4 [file Supplementaryfile3.docx]

**Method**

***Molecular docking of small molecular compounds targeting key CARGs***

To further explore potential therapeutic agents targeting the key CARGs, molecular docking was performed using the Molecular Operating Environment (MOE) software in combination with the FDA-approved small molecule drug library from the ZINC database (https://zinc.docking.org/catalogs/fda/). The 3D molecular structures of the key CARGs were predicted using AlphaFold (https://alphafold.ebi.ac.uk). The missing hydrogen atoms in the 3D structure were added using the MOE software to optimize the structure. In the molecular docking results, compounds with the top three highest S-scores and hydrogen bond binding energies between the receptor and ligand > -5 kcal/mol were considered potential binding drugs for the protein conformation.

**Result**

***Molecular docking of small molecular compounds targeting the key CARGs***

Three top small molecule candidate drugs targeting the three key CARGs were identified through molecular docking using MOE software. The three top small-molecule docking compounds for Col5a1 are as follows: ZINC000009212425 (S = –8.3503; hydrogen bond binding energy = –11.1 kcal/mol) (Figure S3 A); ZINC00000158572 (S = –8.0213; hydrogen bond binding energy = –11.5 kcal/mol) (Figure S3 B); ZINC000011616925 (S = –8.0065; hydrogen bond binding energy = –12.4 kcal/mol) (Figure S3 C). The three top small-molecule docking compounds for Col6a1 are as follows: ZINC000019632618 (S = –7.6844; hydrogen bond binding energy = –6.9 kcal/mol) (Figure S3 D); ZINC000002598638 (S = –7.6616; hydrogen bond binding energy = –5.7 kcal/mol) (Figure S3 E); ZINC000000537791 (S = –7.6537; hydrogen bond binding energy = –21.2 kcal/mol) (Figure S3 F). The three top small-molecule docking compounds for Fbn1 are as follows: ZINC000003830512 (S = –6.8091; hydrogen bond binding energy = –6.0 kcal/mol) (Figure S3 G); ZINC000001530939 (S = –6.3748; hydrogen bond binding energy = –8.0 kcal/mol) (Figure S3 H); ZINC000003799072 (S = –6.3435; hydrogen bond binding energy = –8.4 kcal/mol) (Figure S3 I).

**Figure S3. Molecular docking of small molecular compounds targeting key CARGs**

(A) Molecular docking model of the small-molecule drug ZINC000009212425 with Col5a1. (B) Molecular docking model of the small-molecule drug ZINC00000158572 with Col5a1. (C) Molecular docking model of the small-molecule drug ZINC000011616925 with Col5a1. (D) Molecular docking model of the small-molecule drug ZINC000019632618 with Col6a1. (E) Molecular docking model of the small-molecule drug ZINC000002598638 with Col6a1. (F) Molecular docking model of the small-molecule drug ZINC000000537791 with Col6a1. (G) Molecular docking model of the small-molecule drug ZINC000000537791 with Fbn1. (H) Molecular docking model of the small-molecule drug ZINC000001530939 with Fbn1. (I) Molecular docking model of the small-molecule drug ZINC000003799072 with Fbn1.
